# Edetate Disodium-based Treatment in a Woman with Diabetes and Critical Limb Ischemia Scheduled for Lower Extremity Amputation

**DOI:** 10.7759/cureus.6142

**Published:** 2019-11-13

**Authors:** Francisco Ujueta, Carlos Vozzi, Lara Vozzi, Gervasio Lamas

**Affiliations:** 1 Internal Medicine, Mount Sinai Medical Center, Miami Beach, USA; 2 Cardiology, Instituto Vozzi, Rosario, ARG; 3 Cardiology, Mount Sinai Medical Center, Miami Beach, USA

**Keywords:** peripheral artery disease, critical limb ischemia, extremity amputation, chelating agent, ethylenediaminetetraacetic acid (edta), cadmium

## Abstract

Historically, it is underappreciated that women undergoing amputation for critical limb ischemia (CLI) are older, more severely ill, and have a poorer prognosis than men. Epidemiological studies have shown an association between environmentally acquired vasculotoxic metals, coronary events, and peripheral artery disease. In this paper, we describe an elderly woman with CLI referred for primary amputation underwent edetate disodium-based treatment, known to reduce toxic metal burden, as a final option for limb salvage.

## Introduction

Critical limb ischemia (CLI) carries a mortality rate of 24% at one year and 54-60% at five years, a mortality more severe than most cancers [1]. It is underappreciated that women undergoing amputation for CLI are older, more severely ill, and have a poorer prognosis than men [2]. Epidemiological studies have shown an association between environmentally acquired vasculotoxic metals, coronary events, and peripheral artery disease [3-5]. Edetate disodium has been used for the treatment of atherosclerosis for over 60 years [6-7]. The trial to assess chelation therapy (TACT) demonstrated a possible benefit in patients with diabetes and peripheral artery disease (PAD) [7-8]. We review a case of an elderly woman with CLI, referred for primary amputation, underwent edetate disodium-based treatment, known to reduce toxic metal burden, as a final option for limb salvage.

## Case presentation

**Figure 1:**
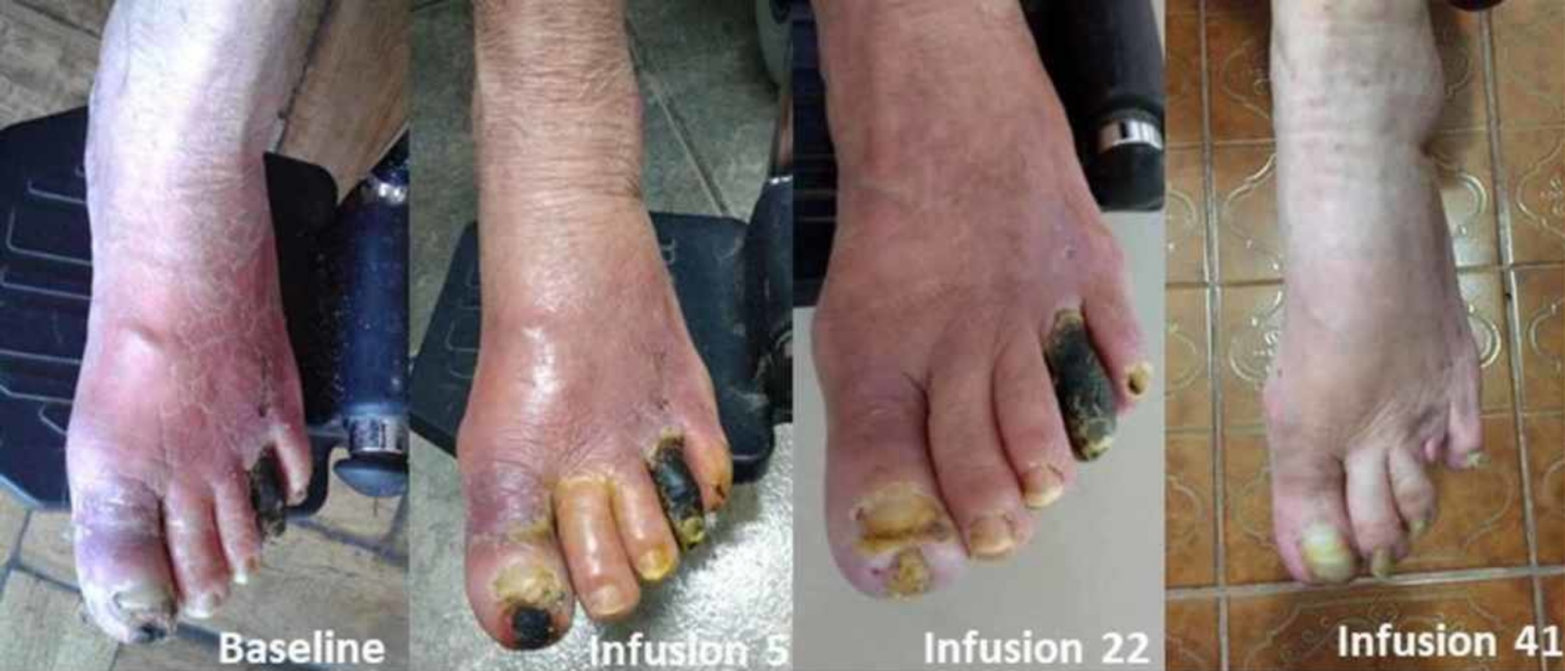
Evolution of dry gangrene Initial presentation and evolution of dry gangrene during treatment with edetate disodium-based infusions.

An 81-year-old female with diabetes was seen at our clinic for a second opinion because of digital gangrene and persistent ischemic rest pain, limiting her ability to walk and sleep. She had been offered a below-knee amputation. Relevant physical findings included bilaterally absent infrapopliteal pulses, with dry gangrene of the left first, fourth and fifth toes, and rubor/erythema (Figure 1). Her blood pressure was 130/80 mmHg, blood sugar 99 mg/dL, and serum creatinine was 0.52 mg/dL with an estimated glomerular filtration rate (GFR) of 120.3 mL/min per 1.73 m^2^. The patient was admitted with a diagnosis of critical left limb ischemia (CLI). Treatment with antibiotics and pain medication was started immediately.

Past medical history

She reported type II diabetes, hypertension, hyperlipidemia, aortic valve replacement (1996) and previous ischemic stroke. She never smoked. Her medications at baseline included aspirin 150 mg, candesartan 8 mg, metformin 850 mg, amlodipine 5 mg, warfarin, rosuvastatin 10 mg, omeprazole 20 mg, levomepromazine 25 mg.

Investigations

**Figure 2:**
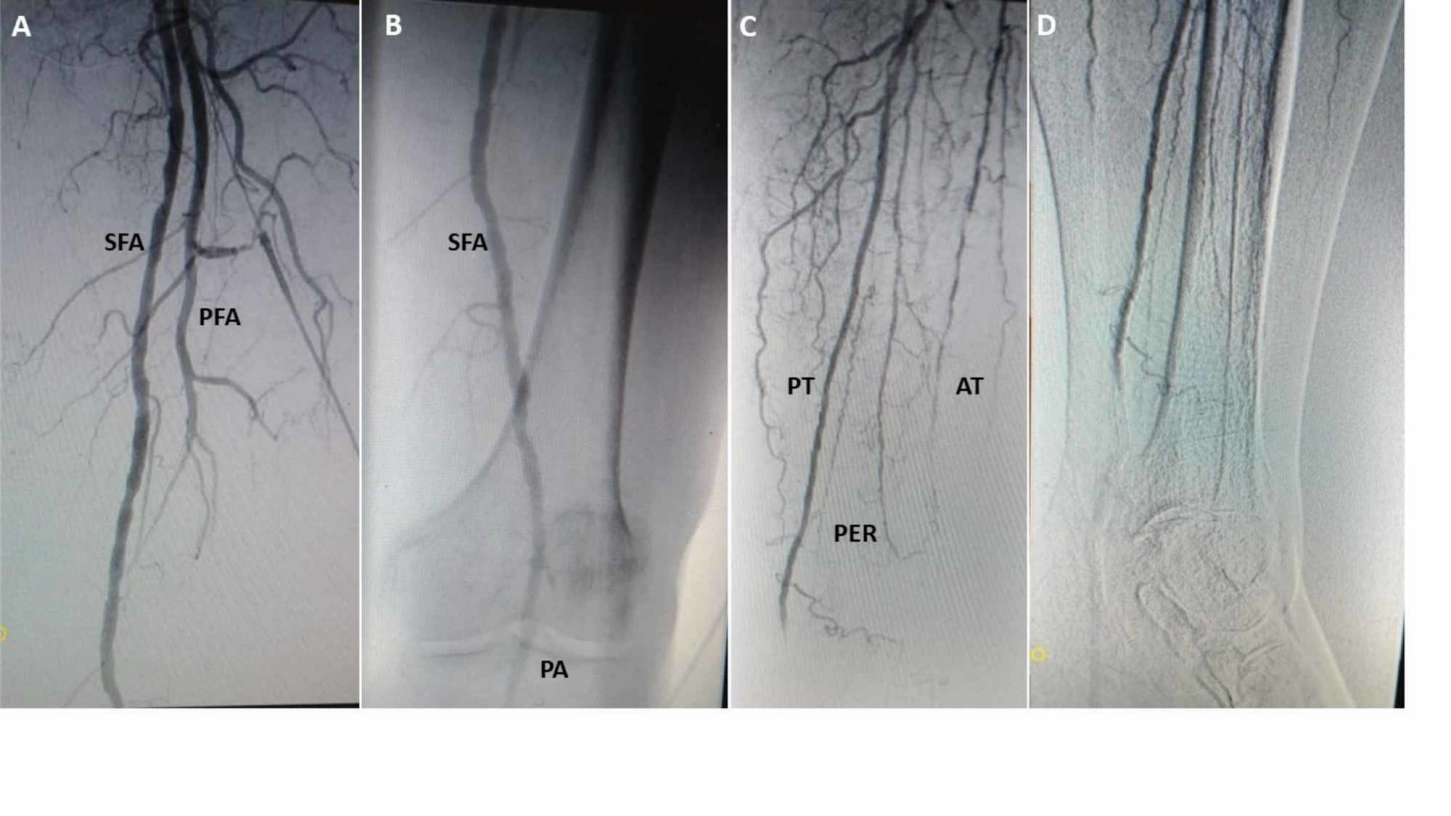
Left lower extremity angiogram at baseline (A) Left superficial femoral (SFA) and profunda arteries (PFA). (B) Patent superficial femoral (SFA) and popliteal (PA). (C) Posterior tibial (PT). Diffuse disease in anterior tibial (AT) and peroneal (PER). (D) Severe disease posterior tibial, no visualization of plantar arteries.

Arterial duplex and angiogram were performed during the initial hospitalization. Left lower extremity arterial duplex demonstrated calcified plaques in the common and superficial femoral, popliteal, anterior and posterior tibial and dorsalis pedis. Monophasic flow was detected below the knee. A left lower extremity angiogram demonstrated diffuse lesions in the suprapatellar vessels. Infrapopliteal arteries disclosed diffuse disease with distal anterior and posterior tibial artery occlusions, as well as multifocal peroneal disease. Vascular flow was absent in the pedal-plantar loop (Figure 2).

Differential diagnosis

The differential diagnosis included diabetic foot or peripheral artery disease (PAD) with CLI. Her prior vascular physician had recommended below-the-knee amputation due to diffuse disease and poor distal arterial beds.

Management

Based on prior results of safety [6] and potential for benefit [7-8], we offered her a treatment regimen of 40 intravenous infusions of 3 g of edetate disodium-based chelation as prepared in the trial to assess chelation therapy (TACT). Infusions included up to 3 grams of edetate disodium adjusted downward based on creatinine clearance. All infusions also contained 2 g of magnesium chloride, 100 mg of procaine HCL, 2500 U of unfractionated heparin, 7 g of ascorbate, 2 mEq of KCL, 840 mg of sodium bicarbonate, 250 mg of pantothenic acid, 100 mg of thiamine, 100 mg of pyridoxine, in a volume of 500 mL. Infusions were given biweekly for the first 10 weeks and then weekly for the remaining 20 weeks, including daily oral minerals and multivitamin supplements. The vascular surgery consultant agreed with this final savage course of treatment.

Clinical course

At the initial hospitalization antibiotics were started for cellulitis. The patient received seven days of ciprofloxacin and clindamycin intravenously as an inpatient and discharged to complete four days of levofloxacin. She was readmitted three days after hospital discharge due to an allergic reaction secondary to oral levofloxacin, treated with antihistamines and discharged without antibiotic treatment with a recommendation for lower extremity amputation.

**Figure 3:**
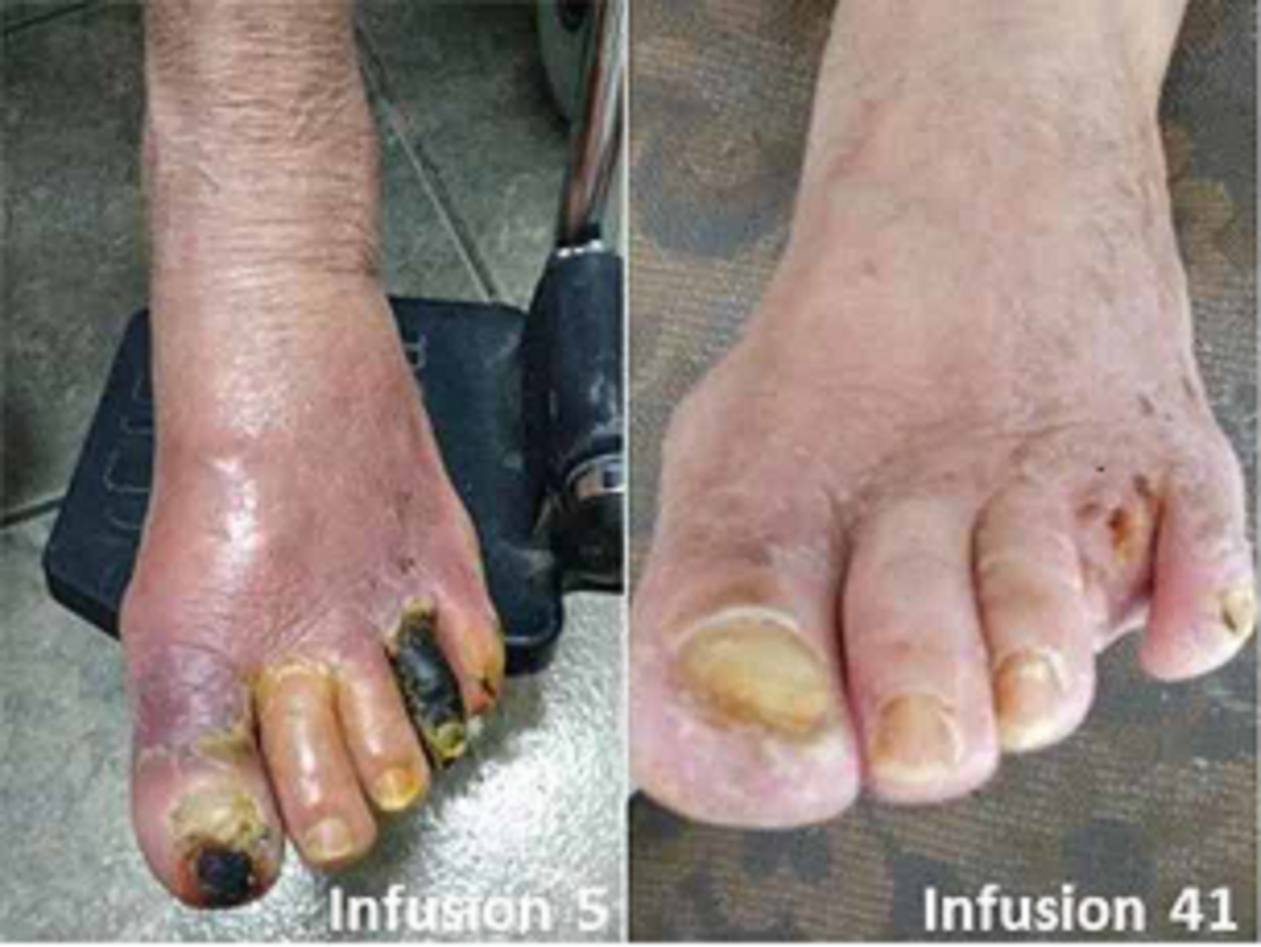
Wound healing of dry gangrene of left hallux

Infusions with edetate disodium began two days after the initial consultation at Instituto Vozzi and 16 days after her initial hospital admission. After the second infusion, rest pain improved, enabling the patient to sleep. At infusion 15, skin color in the left lower extremity improved with only the fourth toe remaining gangrenous (Figure 1). After the 20th infusion she was able to ambulate at home for the first time in a year. Physical examination of the lower extremity at infusion 41, revealed complete healing of the hallux (Figure 3) and improved color and temperature of the left foot.

Because of marked improvement the patient elected to continue maintenance infusions twice per month for 10 additional infusions totaling 50. Although there was no instrument-based measure of quality of life, the patient, upon completion of 47 infusions, 337 days after first presentation for amputation, was pain free, and expressed, “I have my feet again.” There were no treatment-related adverse events. Her serum creatinine at infusion 40 was 0.62 mg/dL with an estimated GFR of 97.9 mL/min per 1.73 m^2^. A urinary tract infection developed which resolved with antibiotic treatment between days 112 and 116. There were otherwise no changes made to baseline medications. The 4th toe painlessly auto amputated after the 40th infusion (Figure 1). Using the same technique and operator compared with baseline, arterial duplex studies performed at infusion 43 demonstrated improved flow in the left AT and PT.

Follow-up

Because of her positive response, the patient has continued maintenance treatments administered twice per month. Now nearly a full year since she was scheduled for amputation, she continues pain-free, walking without assistance, and performing her activities of daily living. She has not required any vascular interventions.

## Discussion

Although men undergo more non-traumatic, vascular-related amputations of the lower extremities, women are usually more severely ill and affected at a more advanced age [6]. It is underappreciated that women tend to have a significantly shorter (1.38 times) post-operative lifetime (95% CI, 1.11-1.72, p = 0.04) after below-the-knee amputation [2]. In 2013, we reported a benefit of edetate disodium-based therapy in patients with a prior myocardial infarction (MI) [7]. A prespecified analysis of patients with diabetes demonstrated a large effect size, with a 41% relative risk reduction of recurrent cardiovascular events (p = 0.0002), including a 43% relative risk reduction in all-cause mortality (p = 0.011) [9]. A non-pre-specified data derived analysis of patients with prior MI, diabetes, and self-reported PAD demonstrated a relative risk reduction of 48% (p = 0.0069) of the TACT primary endpoint compared with placebo [8].

This case report suggests that edetate disodium-based chelation is biologically active and may have a role as adjunct therapy in ‘no-option’ CLI patients [6]. Upon completion of her regimen the patient had avoided a major amputation, regained unassisted ambulation, and maintained her independence. The patient completed all infusions without toxicity.

The data relating low-level toxic metal exposure and vascular disease are robust, suggesting that for some metals, such as lead, there is no safe lower limit. Lanphear et al. published that within “low” lead levels, those in the highest tertile had the greatest cardiovascular mortality [3]. Tellez-Plaza et al. reported that urine cadmium is associated with PAD [4]. Zhuang et al. analyzed the NHANES data for predictors of PAD and found cadmium was one of only four independently predictive variables [5]. Although no measures of urine metals were performed in this clinical case, prior studies have demonstrated, post chelation urine contains about 3000% to 4000% more lead than at baseline and about 700% more cadmium than pre-chelation urine [10]. We have hypothesized that chelation of vasculotoxic metals such as lead and cadmium may lead to improved circulation and limb preservation [8]. We reported urine cadmium levels associated with the severity of PAD [11]. Metals may increase the risk of vascular disease through endothelial dysfunction, inflammation and additional pro-atherosclerotic mechanisms. Thus, decreasing total body levels of metals might plausibly reduce the severity or halt the progression of disease.

Alternate explanations for this woman's case include that she may have simply had severe progressive cellulitis that was ultimately treated successfully. However, this does not explain why by duplex her flow improved, nor her complete recovery nearly a year after therapy. Rare cases of spontaneous recovery of blood flow have been reported, and we can certainly not exclude such an event. Nevertheless, in conjunction with other sporadic case reports, we feel that this adjunct medical regimen should be further studied and selectively offered to similar patients [6].

## Conclusions

These findings suggest a possible role of adjunct edetate disodium-based treatment for diabetic patients scheduled for amputation due to end-stage vascular disease. TACT3a, a double-blind placebo-controlled trial in patients with diabetes and CLI, is now ongoing.
